# Development and Characterization of a Sustainable Bio-Polymer Concrete with a Low Carbon Footprint

**DOI:** 10.3390/polym15030628

**Published:** 2023-01-26

**Authors:** Daniel Heras Murcia, Siham Al Shanti, Fatemeh Hamidi, Jessica Rimsza, Hongkyu Yoon, Budi Gunawan, Mohammed Abdellatef, Mahmoud Reda Taha

**Affiliations:** 1 Department of Civil, Construction & Environmental Engineering, University of New Mexico, Albuquerque, NM 87131, USA; 2 Sandia National Laboratories, MS 0748, 1515 Eubank Blvd. SE, Albuquerque, NM 87123, USA

**Keywords:** polymer concrete, infrastructure materials, carbon footprint, bio-based materials, mechanical characterization

## Abstract

Polymer concrete (PC) has been used to replace cement concrete when harsh service conditions exist. Polymers have a high carbon footprint when considering their life cycle analysis, and with increased climate change concerns and the need to reduce greenhouse gas emission, bio-based polymers could be used as a sustainable alternative binder to produce PC. This paper examines the development and characterization of a novel bio-polymer concrete (BPC) using bio-based polyurethane used as the binder in lieu of cement, modified with benzoic acid and carboxyl-functionalized multi-walled carbon nanotubes (MWCNTs). The mechanical performance, durability, microstructure, and chemical properties of BPC are investigated. Moreover, the effect of the addition of benzoic acid and MWCNTs on the properties of BPC is studied. The new BPC shows relatively low density, appreciable compressive strength between 20–30 MPa, good tensile strength of 4 MPa, and excellent durability resistance against aggressive environments. The new BPC has a low carbon footprint, 50% lower than ordinary Portland cement concrete, and can provide a sustainable concrete alternative in infrastructural applications.

## 1. Introduction

Polyurethane (PU) is a class of polymer that has a wide range of physico-chemical properties. These properties are useful in different fields such as foams, coatings and adhesives, bio-medical sciences, plastic, thermoplastic, elastomers, and recently, the construction industry [1]. PU consists of alternating hard and soft polymer segments. The hard segment is composed of alternating di/polyisocyanate and chain-extender molecules (i.e., diol or diamine), whereas the soft segment is formed from a linear, long-chain diol (polyol). Owing to the thermodynamic incompatibility of the hard and soft segments, phase separation often occurs in PUs [2,3]. Properties of PUs are contingent upon the chemical nature, thermal history, and hydrogen bond in the material. The crystalline structure of the hard segment affects the rheological, mechanical, and thermal properties of the PUs [4,5]. To enhance the properties of PUs, fillers have been considered as a viable alternative [6,7]. Multi-walled carbon nanotubes (MWCNTs) have extensively been utilized in the preparation of polymer nanocomposites due to their high flexibility, low mass density, high aspect ratio, and exceptional mechanical and electrical properties [6,8,9]. Chemically modified MWCNTs, such as carboxyl- (COOH) functionalized graphitized MWCNTs (COOH-functionalized MWCNTs), have been widely used to reinforce polymers due to their high adhesion efficiency [3,10].

Polymer concrete (PC) has appeared as a widely attractive construction material for the last few decades. PC is developed by mixing well-graded aggregate/fillers and a polymer resin that partially or fully substitutes the cement binder. Due to its advantages, PC is usually used in applications that require excellent resistance to aggressive environmental conditions [11], and high resistance to dynamic and cyclic loads (i.e., fatigue) [12]. Compared with conventional cement-based concrete, PC has several advantages, including low permeability, chemical resistance, high ductility, high mechanical strength, and excellent bonding with other types of surfaces [13]. Therefore, PC in infrastructure applications offers high-performing solutions, such as enabling a faster return to service due to its fast-setting time and improved flexibility, versatility, and durability, reducing the need for frequent repairs required by conventional concrete [14]. The main applications of PC in infrastructure include bridge decks, industrial overlays, pavements, wastewater pipes and containers, utility holes, underground communication and transmission line boxes, building façade panels, and machine foundations [15,16,17].

In 1970, URTEK (Finland) developed for the first time commercial PU concrete [18]. PU concrete has been utilized in several applications as a repair material [17,19], coating and sealant for concrete in aggressive environments [20,21], and strengthening material [20,21,22]. Wang et al. [19] studied the flexural and compressive strength of PU concrete as a repair material. Scanning electron microscope (SEM) analysis was conducted to evaluate PU concrete’s microstructure and adhesive properties. Results indicated that the PU polymer bonds firmly with the concrete aggregate. It also showed PU concrete to bond well with cement concrete in repair applications. Hussain et al. [20,21] reported that the flexural performance of the beam strengthened with PU concrete was higher than that of an ordinary concrete beam. A similar result was obtained by Zhang and Sun [22], strengthening concrete beams with PU concrete improved the beams’ ultimate moment capacity and stiffness. Through SEM images, Hu et al. [23] concluded that polyurethane concrete had a self-healing ability. Lei et al. [24] investigated the effect of temperature on polyurethane concrete’s mechanical properties and failure mode. Results showed that as the temperature increases, the crack width and propagation of the specimens at failure decreases, and the failure behavior changes from brittle to plastic failure. Furthermore, the increase in the temperature resulted in a reduction of the compressive strength, splitting tensile strength, and modulus of elasticity.

For its valuable properties, the applications of PU concrete in numerous bridge engineering (airport runways, tunnels, and bridge decks) are gaining considerable significance. PU concrete has been applied in several bridge engineering projects [25,26,27]. Xu et al. [25] evaluated the long-term service of PU concrete used for bridge deck pavement. The long-term service was studied by photothermal coupling aging and thermo-oxidative aging and compared with a modified asphalt mixture. Results showed the PU concrete had better anti-loose performance, low-temperature crack resistance, permanent deformation resistance, water stability, and fatigue resistance than modified asphalt mixtures. Furthermore, Jiang et al. [26] reported that adding 8.5% of PU to concrete increased concrete’s rutting and cracking resistance. Additionally, PU concrete possesses an excellent anti-stripping performance and good resistance against the alkaline solution, which ensures PU concrete pavement’s long service life.

Despite the benefits of PU concrete in the construction industry, PU polymer is well-known to have a very short setting time [28]. As reported, PU concrete starts setting within minutes, and it usually takes 30 min for the final set to occur [28]. The short setting time causes difficulty handling PU concrete in construction. Li and Yu [29] investigated the effect of using castor oil and phosphate ester as retarders on setting time and durability of PU concrete incorporating cement. Results showed that 1% of phosphate ester increased the setting time by 2 h. Moreover, the PU concrete showed better frost, wear, bending fatigue, and UV aging resistance than cement concrete [29].

Recently, studies were directed toward investigating the potential of using bio-based polyurethane aggregate in concrete. Sari et al. [30] studied the performance of using palm oil-based PU to partially replace aggregates from 0% to 3% to produce lightweight concrete. It was reported that as the aggregate replacement with palm-based PU increases, the compressive strength decreases and the thermal conductivity of concrete increases. Nevertheless, limited research has been conducted on characterizing and producing bio-based PU concrete as a structural material [31,32,33]. This research is focused on investigating the potential development of bio-based PU concrete, where bio-based PU is used as the binder in lieu of cement, as a sustainable alternative for reference cement concrete and other PCs in infrastructure applications. This work aims to characterize physically, mechanically, and chemically bio-based PU PC, denoted herein as bio-polymer concrete (BPC).

## 2. Materials and Mix Design

**Figure 1:**
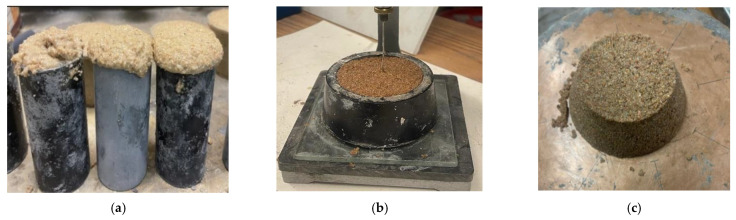
Challenges to producing bio-polymer concrete (BPC) identified through preliminary investigations showing; (**a**) foaming, (**b**) fast setting, and (**c**) zero flowability.

BPC is developed herein using polyol, isocyanate, and medium-graded aggregate. The polyol was Sovermol 750 (BASF, Wyandotte, MI, USA). No cement was used in making the BPC. Sovermol 750 is a bio-based derived polyol with 65–80% renewable natural oil content. Natural-oil polyols are polyfunctional alcohols based on renewable raw materials like rapeseed oil, castor oil, soybean oil and palm kernel oil. Sovermol 750 has the hydroxyl number in the range of 300–330 mgKOH/g, moisture content of less than 0.1%, and acid number of less than 2.0. The isocyanate used was solvent-free, medium-viscosity polymethylene polyphenyl polyisocyanate, Lupranate M70L (BASF, Wyandotte, MI, USA) with NCO content of 31.0 wt.%. Several trial mixes were designed and tested to identify, examine and overcome the challenges of producing BPC, including excessive foaming, fast setting, and zero flowability, as depicted in Figure 1a–c, respectively.

It was suggested that when the mix acidity is increased, foam formation can be stopped due to the blockage of the active –NCO sites of polyisocyanate [2]. It was also shown that acids with the *pK_a_* in the range of 2.8–4.5 will retard rather than prohibiting the polymerization of PU [34]. Therefore, benzoic acid (99.5% purity) with *pK_a_* equal to 4.2 was selected as the retarder for the BPC. The polyol was nano-modified utilizing COOH-functionalized MWCNTs (Cheap Tube Inc., Grafton, VT, USA). Introducing COOH-functionalized MWCNTs to the polymer matrix is expected to improve the rheology of the polymer, compensate the possible strength deterioration due to the use of acid, and enhance the thermal stability of BPC [10,35]. Table 1 presents the main characteristics of MWCNTs in this research.

Table 2 presents the mix design of 1 m^3^ of BPC. The design criteria was that the BPC would reach a setting time of at least 30 minutes and have a 7-day compressive strength of 20.7 MPa (3000 psi). Both criteria are driven by the need to improve the constructability of BPC. Twenty trial mixes with different PU contents (30, 24, 20, 18, and 16 vol.% of the total concrete volume) and benzoic acid dosages (3.0, 4.0, 5.0, and 6.0 wt.% of the total binder content) were carried out. Adding PU at 16 vol.% of the total concrete volume and 4.0 wt.% of the total binder content resulted in the best performance for BPC at both fresh and hardened states. Hence, for the rest of the experiments, the 16 vol.% PU and 4.0 wt.% benzoic acid were selected as the reference mix to investigate the impact of benzoic acid and COOH-functionalized MWCNTs on the behavior of the BPC.

**Figure 2:**
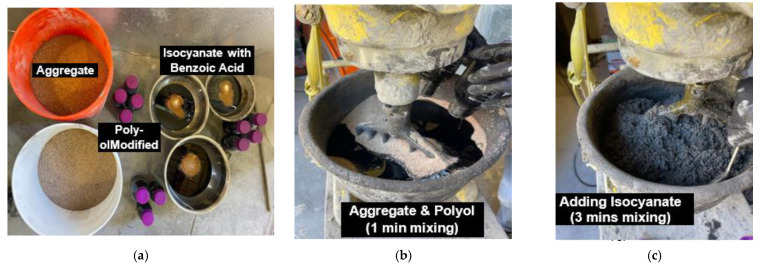
Mixing of BPC. Steps shown from left to right: (**a**) ingredients, (**b**) step 1 mixing, and (**c**) step 2 mixing.

Before mixing, the polyol was nano-modified utilizing COOH-functionalized MWCNTs at an optimum dosage of 0.5 wt.% of binder; the optimum dosage was chosen based on previous work [9]. The dispersion procedure was performed following previous work done by Douba et al. [10]. COOH-functionalized MWCNTs were dispersed in polyol through sonication at 55 °C for 40 min and centrifuged at 2000 rpm for 20 min to remove the nano-particle clusters. At this point, the nanocomposite was left to cool to room temperature for 2 h^.^ After 2 h, BPC’s ingredients were mixed as follows: the aggregate and nano-modified polyol were added to the mixer and mixed at room temperature at a rate of 1500 rpm for 1 min to ensure that the aggregate and polyol were fully and evenly mixed. For safety, the isocyanate and benzoic acid were slowly added and continued for three minutes. The materials and mixing procedure are shown in Figure 2. After the raw materials were sufficiently mixed, the BPC mix was cast in 8-inches (203 mm) height by 4-inches (101 mm) diameter cylinders in three layers and compacted 25 times after pouring each layer. The excess mixture was removed, the surface was leveled by using a scraper, and the specimens were de-molded after 2 h. To examine the effect of curing temperature on the polymerization and mechanical properties of BPC, specimens were air cured at an indoor temperature and heat cured in an oven at 65 °C for 7 days before testing. Air-cured BPC mixes are denoted BPC-A, while heat-cured BPC mixes are denoted BPC-H.

## 3. Experimental Methods

**Figure 3:**
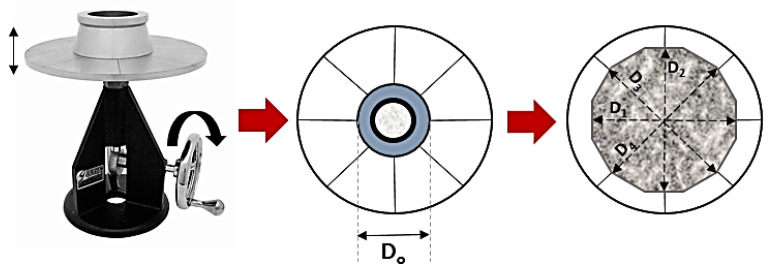
Schematic representation of flow table test. Figure shows the original base diameter (D_0_) and the four measured diameter of concrete after flow (D_1_, D_2_, D_3_ and D_4_).

Immediately after mixing, flowability and initial setting time of the BPC were evaluated. The flow table test at time zero was measured as per ASTM C230-14 [36]. The flow table test apparatus had a cone height of 2-inches (50 mm), and a varying diameter of 3-inches (76 mm) on the top to 4-inches (101 mm) on the bottom. The flowability was calculated as an average increase of four measured diameters (D_1_–D_4_) and expressed as a percentage of the original base diameter (D_0_). A schematic representation of the flow table test is shown in Figure 3. Initial setting time testing was conducted using the standard test method for hydraulic cement by Vicat Needle ASTM C191-18a [37]. Only one sample was tested for both the flow table and the setting time tests as per ASTM standards [36,37].

The mechanical performance of BPC, including the modulus of elasticity, Poisson’s ratio, compressive, and splitting tensile strength, was evaluated. All mechanical testing was carried out at 7 days of age on cylindrical specimens of 8-inch (203 mm) height and 4-inch (101 mm) diameter. For each curing method, four specimens were tested. The static modulus of elasticity and Poisson’s ratio tests were performed as per ASTM C469-14 [38]. A Forney QC-400-D (Forney LP, Zelienople, PA, USA) VFD automatic testing machine with a resolution of 1 lbf (4.45 N) and a maximum capacity of 400 kips (1779 kN) was used to determine both the static modulus of elasticity and Poisson ratio. A ramp rate of 145 psi/s (1 MPa/s) was applied^.^ The compressive and splitting tensile strength tests were conducted following ASTM C39 [39] and ASTM C496 [40]. Both tests were conducted using a Tinius Olsen (Tinius Olsen, Horsham, PA, USA) model 55,922 universal testing machine (UTM) with a resolution of 1 lbf (4.45 N) and a maximum capacity of 400 kips (1779 kN). A displacement rate of 1 mm/min (0.039 in/min) was used during the tests. The splitting tensile strength was calculated using Equation (1).

(1) $$f_{t}=\frac{2P}{\pi DL}$$

where *f_t_* is the split tensile strength, *P* is the applied load, *D* is the diameter of the specimen, and *L* is the length of the specimen.

The durability of the BPC was assessed by examining weight loss after exposure to different mediums as per ASTM D543-14 [41]. The 4 weighed specimens of BPC and 4 weighted specimens of ordinary concrete were placed into the hydrochloric acid (HCL) 37% at ambient temperature. The weight of the specimens was measured in 4-day intervals over 20 days. The acid solution was changed regularly to keep the medium’s pH below 7. After every 4 days, the samples were removed and washed with distilled water, dried in the oven at 115 °C and weighed using a precision balance.

Microstructure characterization of BPC using a SEM was used to examine the microstructural features of air and heat-cured BPC. Fractured BPC specimens were examined using a VEGA3 thermionic emission SEM system by TESCAN (Brno, Czech Republic). To improve conductivity, the specimens were sputter-coated with gold/palladium (Au/Pd). The electron beam energy used for all readings was 20 keV. The magnification varied from 400× to 1150× depending on the features to be detected.

The carbon footprint analysis was performed to assess the greenhouse gas (GHG) emissions associated with manufactured BPC. The carbon footprint was evaluated by multiplying the quantity of each material in the mix design by its CO_2_ equivalent using CCalC2 toolbox developed at the University of Manchester [42]. The results of BPC’s carbon footprint analysis were compared with a typical cement concrete mix.

Finally, chemical characterization of biopolymer PU on different polymer specimens was conducted, including pristine PU, PU polymer with 4 wt.% of benzoic acid, and PU nanocomposite containing 4 wt.% of benzoic acid and 0.5 wt.% of COOH-functionalized MWCNTs. We point out that chemical characterization tests used here are those typically used with polymers rather than those used with cement as the new BPC concrete has no cement. The goal of the chemical characterization was to (i) determine the role of benzoic acid in eliminating the foaming and short setting time of pristine PU polymer, (ii) elaborate on the role of reduced/delayed crosslinking density on the thermal properties of the polymer specimens, and (iii) investigate the role of addition of COOH-functionalized MWCNTs on the crosslinking density and thermal stability of the polymer specimens.

To examine the thermal transitions of PU specimens, as well as measure the degree of crosslinking in the polymer specimens quantitatively, differential scanning calorimetry (DSC) measurements were carried out using a Discovery DSC 25 instrument from TA Instruments (New Castle, DE, USA) operated under a nitrogen environment. Cured and uncured PU samples (5–10 mg) were heated up from 50 °C to 300 °C at a constant heating rate of 10 °C/min, held at 300 °C for 10 min and then cooled down to 50 °C following the same rate. The remaining crosslinking capability in the PU samples was determined by the degree of crosslinking, *X_SX_*, using Equation (2) [43].

(2) $$X_{Sx}=\frac{\Delta H_{S0}-\Delta H_{Sx}}{\Delta H_{S0}}$$

where Δ*H_Sx_* is the crosslinking reaction enthalpy of each test sample, and Δ*H_S_*_0_ is the reaction enthalpy of the reference uncured PU sample. The degree of crosslinking in the PU specimens was investigated qualitatively using the swelling index test. Three-disc specimens (~5 g each) were placed in 30 mL of toluene (99.5% purity, Fisher Scientific, Waltham, MA, USA) and kept at ambient temperature for 2 h. The solvent was then decanted, and the specimens were surface dried by short contact with the filter paper to remove the liquid solvent adhering to the surface of the specimen. Promptly, the weight of the swollen polymer was determined using Equation (3).

(3) $$\%\mathrm{Weight}\;\mathrm{Gain}=\frac{M_{2}-M_{1}}{M_{1}}\times 100\;\;\;\;\;\;\;M_{2}\geq M_{1}$$

where *M*_1_ is PU weight before placement in the solvent, and *M*_2_ is PU weight after placement in the solvent.

Fourier transform infrared spectroscopy (FTIR) analysis and thermogravimetric analysis (TGA) were conducted to identify the differences between the functional groups of PU specimens and the mechanism of material decomposition, respectively_._ Nicollet IS50 spectrometer (Thermo Fisher Scientific Inc., Waltham, MA, USA) was utilized to perform the attenuated total reflectance infrared (ATR-FTIR) spectroscopy. The test was conducted using a scanning range of 4000–500 cm^−1^, a resolution of 4 cm^−1^, and a number of sample scans equal to 32. TGA was conducted on a TA550 thermogravimetric analyzer from TA Instruments operated under a nitrogen environment. The specimens were heated up from 50 °C to 450 °C at a constant heating rate of 10 °C/min, and the relative mass loss of the samples was recorded.

## 4. Results and Discussion

### 4.1. Flowability and Setting Time

**Figure 4:**
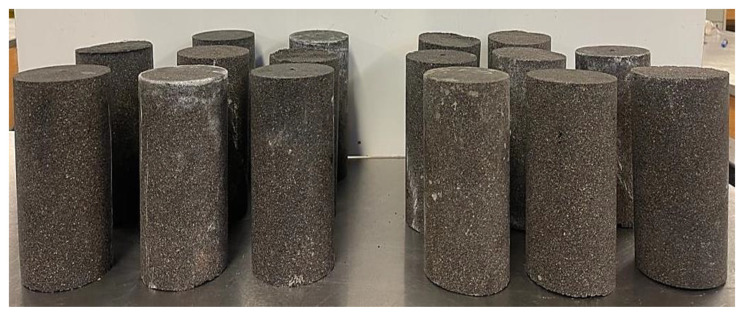
BPC concrete specimens produced showing no foaming in specimens.

BPC maintained the shape of the mold, showing a high stiffness, thus, resulting in a 0% flowability as presented in Table 3. All efforts to improve the flowability of BPC were unsuccessful. Moreover, BPC had a relatively short setting time where it fully hardened after 30 min from the start of mixing. This phenomenon is due to the fast, highly exothermic polymerization of PU, and competing reactions between polyisocyanate and polyol to produce a crosslinked PU [28,44]. Benzoic acid showed an insignificant effect in controlling the rate of polymerization of PU. Nevertheless, introducing acid to BPC was sufficient in eliminating the excessive foaming arising from the reaction between polyisocyanate components and water molecules in the air at room temperature, generating CO_2_ [44,45]. Figure 4 shows the concrete specimens produced herein for characterization without any foaming. Similar results for setting time were reported by Hong et al. [28]. However, Li and Yu [29] reported a slightly longer initial setting time of 40 min and a final setting time of 70 min for PU-cement concrete.

### 4.2. Mechanical Properties

All mechanical testing was performed on four different specimens for each curing condition (air and heat curing). The Student’s *t*-test (*t*-test) was applied to examine the statistical significance between the means. A 95% confidence interval was used (α = 0.05) for all the *t*-tests. Table 4 represents the mean hardened density, modulus of elasticity, and Poisson’s ratio for BPC mixes for each curing condition; air (BPS-A) and heat (BPC-H) cured.

BPC-A and BPC-H showed similar densities with a mean value of 1830 kg/m^3^ ± 45 kg/m^3^ and 1858 kg/m^3^ ± 36 kg/m^3^, respectively. Therefore, thermal curing did not affect the density of BPC. Results were in agreement with PU concrete densities reported in the literature [21,46]. It is worth noting that the density of PU concrete is highly affected by the reaction of water with polyol and polyisocyanate, as a very small addition of water could produce foamed composite and air bubbles which play an essential role in forming the PU densities. PU concrete was found to have a lower density than other commonly used PC [47,48], and conventional cement concrete [49], which implies that BPC could be used as a lightweight material in infrastructural applications.

The modulus of elasticity and Poisson’s ratio of air-cured BPC were 9.3 GPa ± 0.9 GPa, and 0.24, respectively, while for heat-cured BPC, the elasticity modulus slightly increased to have an elasticity modulus of 9.9 GPa ± 0.9 GPa and a Poisson’s ratio of 0.26. The effect of thermal curing on the modulus of elasticity was not statistically significant (*t*-test, α = 0.05). Both curing methods showed modulus of elasticity values similar to those reported in other PCs and lower than normal-strength concrete [49,50]. Moreover, Poisson’s ratio was similar to that of PC and higher than ordinary concrete [49,50]. The low modulus of elasticity and high Poisson’s ratio is an advantage when using BPC for PC, which can reduce the tensile stress buildup from restrained shrinkage, suppressing the reflective cracking, known as the cracks occurring in an overlay material (i.e., tile) in contact to the concrete due to the stresses induced by the shrinkage of the concrete.

**Figure 5:**
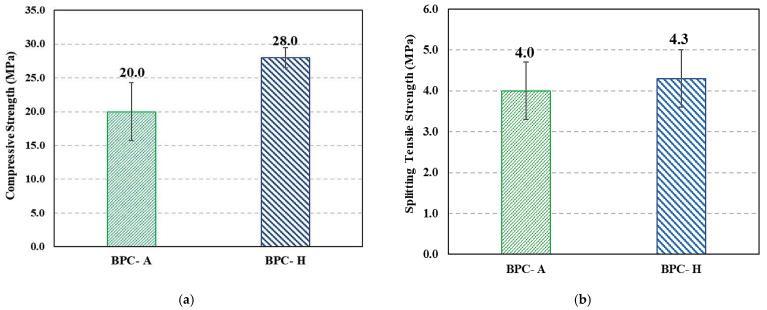
Mechanical strength results: (**a**) compressive strength, and (**b**) splitting tensile strength. Values shown on top of the bar graph represent the mean value.

Figure 5 shows the compressive and tensile strengths of air (BPS-A) and heat-cured (BPS-H) BPC. Heat-cured specimens had higher compressive and tensile strengths than air-cured specimens. While the effect on thermal curing on compressive strength is statistically significant, the effect on the tensile strength was not statistically significant (*t*-test, α = 0.05).

**Figure 6:**
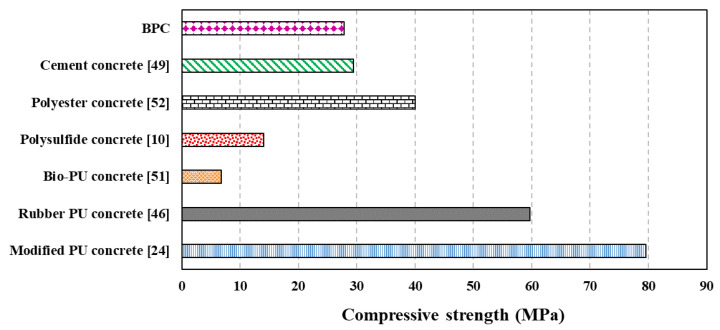
BPC compressive strength comparison with literature.

**Figure 7:**
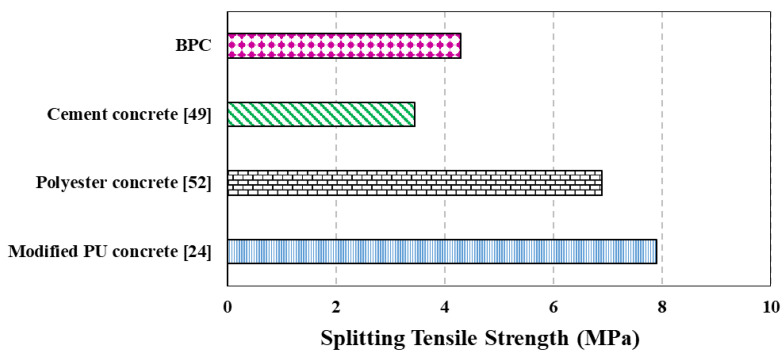
BPC splitting tensile strength comparison with literature.

Figure 6 and Figure 7 show a comparison between produced BPC and previous studies conducted on PU concrete, commonly used PC, and reference cement concrete in terms of compressive and tensile strength, respectively. BPC had a lower compressive strength than PU concrete prepared with rubber waste and cement [46] and modified PU concrete with toluene diisocyanate trimer and hexamethylene diisocyanate trimer [24]. However, the produced BPC in this study had higher compressive and tensile strength values than concrete with recycled ceramic aggregate and bio-based PU binder [51]. Due to the low reactivity of vegetable oils that hinders their polymerization, BPC usually exhibits lower mechanical properties compared to other PCs. However, in this study, the use of COOH-functionalized MWCNTs compensated for the loss of compressive strength resulting from using a bio-based polyol and the addition of acid. Both compressive and tensile strengths of BPC were comparable to normal strength concrete [49] and commonly used PC [10,52]; confirming their potential to be used as a sustainable alternative to PC and cement concrete in infrastructural applications.

### 4.3. Durability

**Figure 8:**
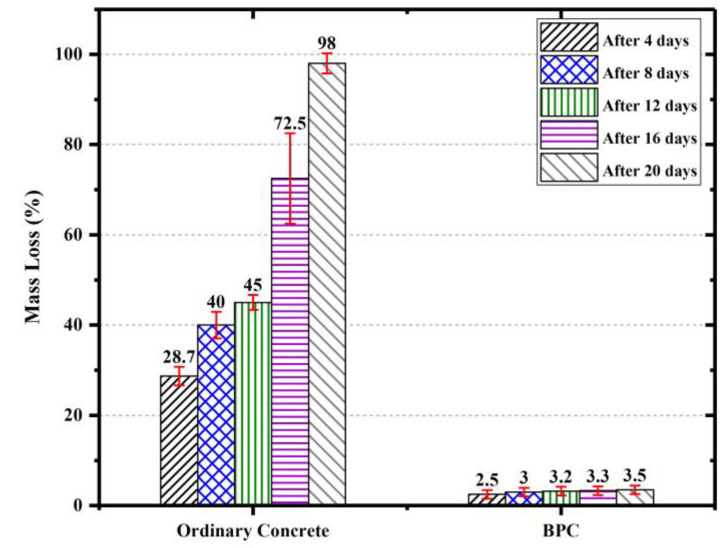
Weight loss of ordinary and BPC exposed to hydrochloric acid (HCL). Values shown above the bars represent the mean values.

Figure 8 represents the results of the durability of BPC and ordinary concrete against hydrochloric acid (HCL). During the whole exposure duration, the weight loss for the BPC was significantly less than that for the ordinary concrete. After 12 days of exposure to the HCL, the ordinary concrete lost almost half of its initial weight, while the BPC only underwent 3% weight loss due to chloride ingress. After 16 days, the ordinary concrete lost almost two-thirds of its initial weight and the specimens turned out to be very loose, while for the BPC, the weight loss percentage remained under 5%. As was observed, 20-day exposure to HCL led to the destruction of the ordinary concrete specimens. However, the mean mass loss for the BPC specimens remained under 5%. The results clearly show that the BPC outperformed the normal concrete’s durability in extreme environmental exposure. Submerging the ordinary concrete specimens in HCL will lead to damages induced by removing the surface coverage and intensive dissolution of cement within the concrete structure into the acid environment which happens because the chloride ions preferentially coordinate with the calcium ions [53]. Due to the lack of cement binder within the bio-based PU concrete, the chloride degradation is much lower than that for ordinary concrete.

### 4.4. Microstructural, Chemical and Carbon Footprint Analysis

**Figure 9:**
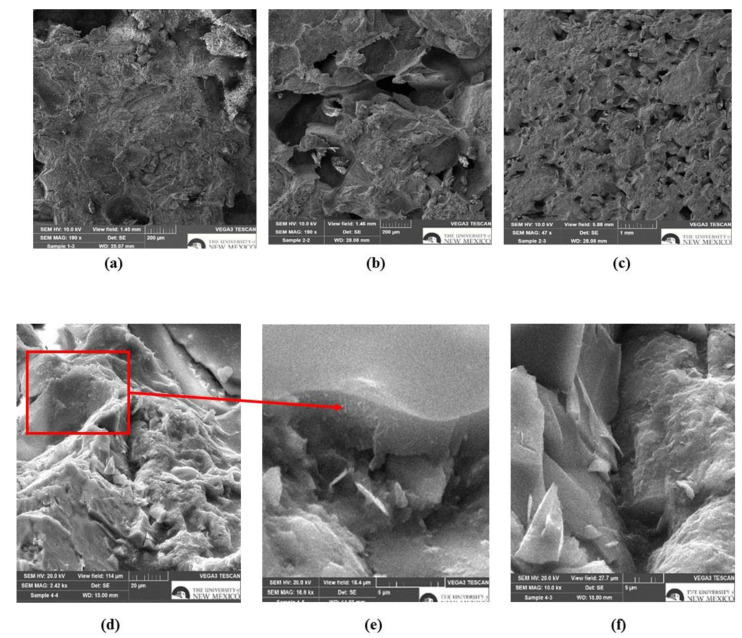
Scanning electron microscope (SEM) images of BPC: (**a**) PU concrete, (**b**,**c**) porous structure of PU containing benzoic acid, (**d**) compact structure of PU concrete with benzoic acid and MWCNTts, (**e**) presence of MWCNTs in the voids, and (**f**) presence of MWCNTs in the cracks.

SEM images of fractured BPC specimens are presented in Figure 9. Figure 9a shows the microstructure of PU concrete without addition of acid and/or nanoparticles, while Figure 9b,c indicate the presence of more voids within the PU concrete due to the addition of benzoic acid. As mentioned before, benzoic acid reduces the crosslinking density, and subsequently hinders formation of compact microstructure. Figure 9d represents sufficient dispersion of functionalized COOH-MWCNTs within the PU matrix. Functionalized COOH-MWCNTs were detected in both voids (Figure 9e) and bridging the cracks (Figure 9f). The presence of functionalized COOH-MWCNTs in voids and between cracks indicated that the surface functionalized MWCNTs rendered formation of a dense microstructure owing to the enhanced crosslinking density and a micro-filling behavior in the matrix. This led to reduction of the cracks’ width, thus, improving the mechanical performance and durability of the BPC.

**Figure 10:**
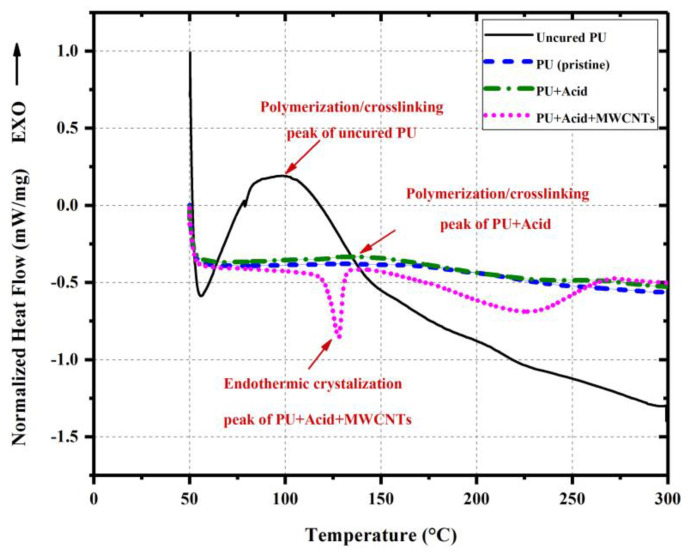
Differential scanning calorimetry (DSC) curves of PU specimens.

The chemical characterizations of the bio-based PU provide significant insight into the behavior of BPC. The DCS thermograms of PU specimens are depicted in Figure 10. For the uncured PU specimen, the broad sharp exothermic peak in the range of 60–140 °C is representative of the polymerization/crosslinking reaction. The equivalent enthalpy of polymerization/crosslinking reaction is approximately 254 J/g (Δ*H_S_*_0_). For the cured pristine PU and PU containing 4 wt.% benzoic acid and 0.50 wt% COOH-functionalized MWCNTs, this peak almost disappeared, showing the roughly fully polymerized/crosslinked specimen (~100%). However, for the PU specimen containing 4.0 wt.% benzoic acid, a small exothermic peak in the range of 125–160 °C can be observed, which corresponds to the crosslinking enthalpy of 6 J/g (Δ*H_Sx_*). Thus, the degree of crosslinking for the PU containing 4 wt.% benzoic acid is equal to 97%, meaning less polymerization/crosslinking degree due to the presence of the acid. However, the difference between the DSC thermograms of pristine PU and PU containing 4.0 wt.% benzoic acid was marginal, indicating the complete reaction of acid moieties with the PU system. An endothermic peak appeared at around 130 °C for the PU nanocomposite compared to other specimens. The appearance of such an endothermic peak indicates that some fractions of the hard segments present in the PU matrix participate in the regular crystalline arrangements due to the presence of chemically treated MWCNTs, which was not observed for the pristine PU and PU containing 4.0 wt.% benzoic acid. The findings align with other researchers’ findings reported in the literature [2,7].

**Figure 11:**
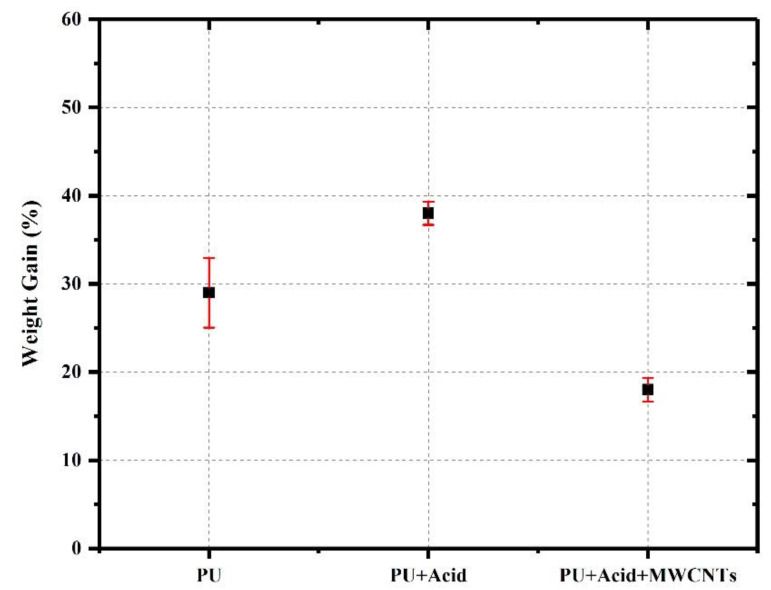
Weight gain percentage of polymer specimens due to solvent intake in the solvent swelling test.

The results of solvent swelling tests of PU specimens are shown in Figure 11. The presented values are the mean value of three identical specimens from the same polymer mixture. The addition of benzoic acid led to 9% more weight gain for the PU sample containing acid compared to the pristine PU sample. In comparison, the addition of COOH-functionalized MWCNTs along with the benzoic acid reached 11% less weight gain compared to pristine PU. The results indicate reduced crosslinking density due to the addition of benzoic acid to the pristine PU. A plausible reason behind such observation could be attributed to the effect of the acid introduced to the polymerization environment. Researchers [54] showed that the reaction kinetics of PU formation depends not only on the reactivity and conditions of the primary reactants but also on the catalysts, which may vary in structure and reactivity. As reported by Han and Urban [55], the equilibrium reaction (Equation (4)) shifts toward the starting reagent side by increasing the acidity. This shift will decrease the concentration of the active anion (component 3: ${\left[R_{2}S_{n}{\left(\mathrm{OCO}\;R^{\prime }\right)}_{2}{\mathrm{OR}}^{\prime \prime }\right]}^{-}$), thus decreasing the reaction rate constant.

(4) $$R_{2}S_{n}\mathrm{OCO}\;R^{\prime }+R^{\prime \prime }\mathrm{OH}{↔}_{K_{2}}^{K_{1}}{\left[R_{2}S_{n}{\left(\mathrm{OCO}\;R^{\prime }\right)}_{2}{\mathrm{OR}}^{\prime \prime }\right]}^{-}+H^{+}$$

Data obtained here are in agreement with the findings of other researchers [2,55] that reported how the introduction of a small dosage of a strong acid, such as HCL, prohibited the formation of PU; while the implementation of a weaker acid, such as acetic acid and benzoic acid, has a less pronounced inhibiting effect.

**Figure 12:**
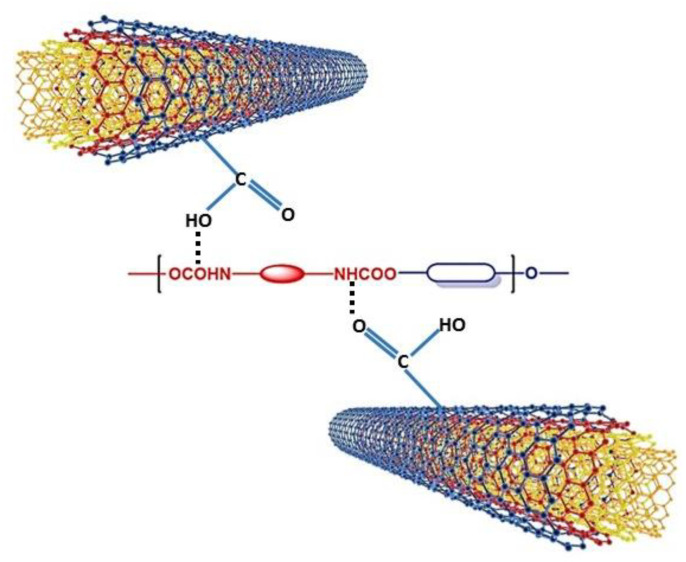
The most probable interaction exists via hydrogen bonding between COOH-functionalized MWCNTs and PU matrix.

Notwithstanding, the addition of COOH-functionalized MWCNTs not only promoted the crosslinking density of the PU specimen containing acid but also led to more crystallinity in the sample. The most probable mechanism by which the COOH-functionalized MWCNTs enhanced the degree of crosslinking is shown in Figure 12. As it can be observed, the COOH-functional groups residing on the surface of the MWCNTs enhance the interfacial interaction with the PU matrix through hydrogen bond formation between the C=O and N-H groups of hard segments of PU chains and COOH groups of COOH-functionalized MWCNTs [3,7]. Both observed effects due to the addition of acid and COOH-functionalized MWCNTs could be attributed to the elimination of the foaming phenomenon in the BPC.

**Figure 13:**
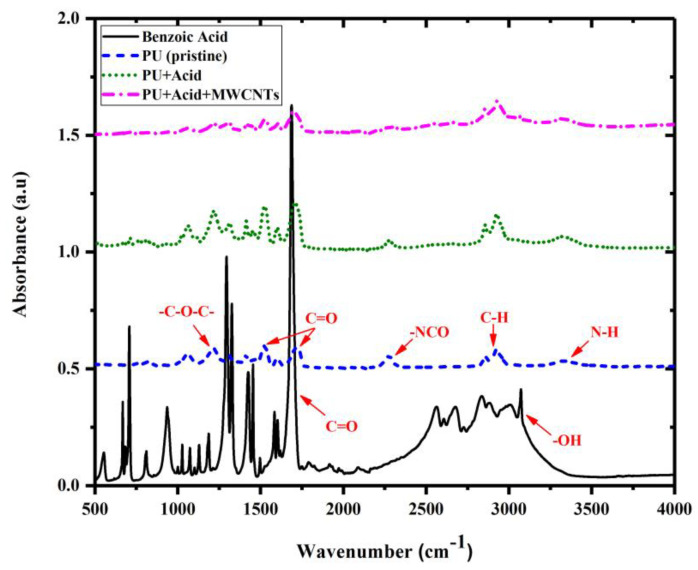
Fourier transform infrared spectroscopy (FTIR) spectrums of benzoic acid and polymer specimens.

Figure 13 represents the FTIR spectrums of PU specimens. PU specimens containing benzoic acid showed a broad peak from 3100 cm^−1^ to approximately 2600 cm^−1^ indicating the acidic –OH stretch^.^ The peak related to the C=O stretch was located at 1670 cm^−1^. Other bands are characteristic of this aromatic carboxylic acid compound [56]. For the pristine PU, the characteristic absorption peaks corresponding to the stretching vibration peak for the –C-O-C- (ether bond) located at 1210 cm^−1^, and the stretching vibration peak for the –NCO (isocyanate groups) showed up at 2250 cm^−1^. The stretching modes at about 1550 and 1725 cm^−1^ are attributed to the hydrogen-bonded and free C=O groups present in urethane linkage (–HN–COO–) of the PU matrix, respectively [57].

**Figure 14:**
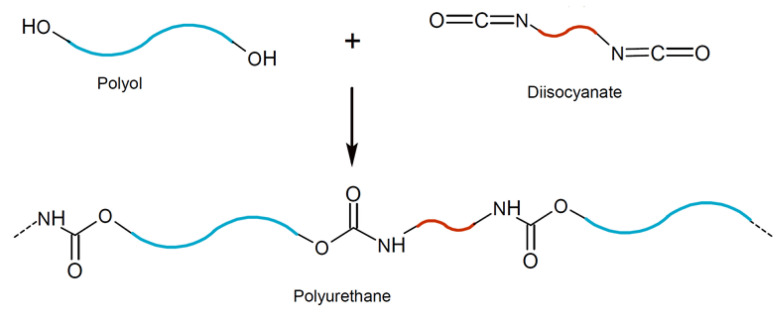
The reaction between polyol (blue) and diisocyanate (red) repeated units leading to the formation of PU.

The reaction between a polyol and diisocyanate is schematically shown in Figure 14. With the addition of benzoic acid, the area under the peaks for the free and hydrogen-bonded C=O, C-H, and N-H groups increased. Increasing the number of free C=O radicals is indicative of a prolonged polymerization/crosslinking between the polyol and diisocyanate, while increasing the number of hydrogen-bonded C=O could be attributed to the elimination of the foaming issue observed in BPC. With the addition of the COOH-functionalized MWCNTs, the intensity of the peaks for both the free and hydrogen-bonded C=O decreased, however, the area under these peaks increased compared to the PU specimens containing only benzoic acid. Moreover, the peak corresponding to the –NCO group disappeared, and the N-H peak intensified compared to the pristine PU specimen. The observed increment in the area under the absorption peaks for the free and hydrogen-bonded C=O reveals the physical adhesion due to the hydrogen bond between both the –NH and C=O group of the PU matrix and the functional COOH group of MWCNTs. In addition, the disappearance of the –NCO absorption peak and amplifying the N-H absorption peak indicate the reaction between the –NH_2_ and –NCO to form a urea bond structure, further polymerization/crosslinking, and formation of a network structure. Bonds formed within the PU matrix due to the addition of surface functionalized MWCNTs confirmed observations through the SEM images (Figure 9) indicating that COOH-functionalized MWCNTs will contribute to the enhancement of the crosslinking density of the PU binder of the BPC. The findings here are in agreement with those reported by [7,58].

**Figure 15:**
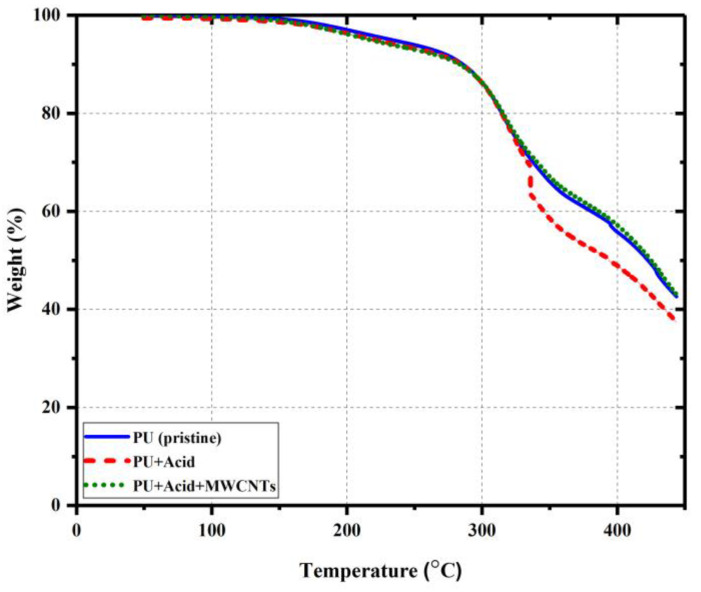
Thermogravimetric analysis (TGA) analysis of polymer specimens.

TGA analysis of PU specimens is presented in Figure 15. The first stages of thermal degradation for all the specimens occurred at around 325 °C accompanied by a 20% loss of the initial weight of each specimen. Up to 340 °C, the thermal stability of all the polymer specimens followed the same trend, which is sensible to not see any significant difference as the degree of polymerization/crosslinking for all three specimens was roughly the same (97% for PU containing 4 wt.% benzoic acid, and almost 100% for the other two specimens). At 340 °C, the thermal decomposition for the PU containing benzoic acid occurred. However, the thermal decomposition of the pristine PU and PU nanocomposite took place at 400 °C, accompanied by losing almost 42% of their initial weight. PU nanocomposite presented a slightly better performance in retaining its weight over the pristine PU. This could be due to the homogeneous dispersion of the COOH-factionalized MWCNTs in the PU matrix, consequently enhancing the thermal conductivity of the PU matrix [56]. Typically, the presence of the COOH-functionalized MWCNTs should have led to better thermal performance for the PU nanocomposite [7]. However, due to the presence of the benzoic acid and its action on the degree of polymerization/crosslinking, no significant difference between the pristine PU and the PU nanocomposite could be traced here.

**Figure 16:**
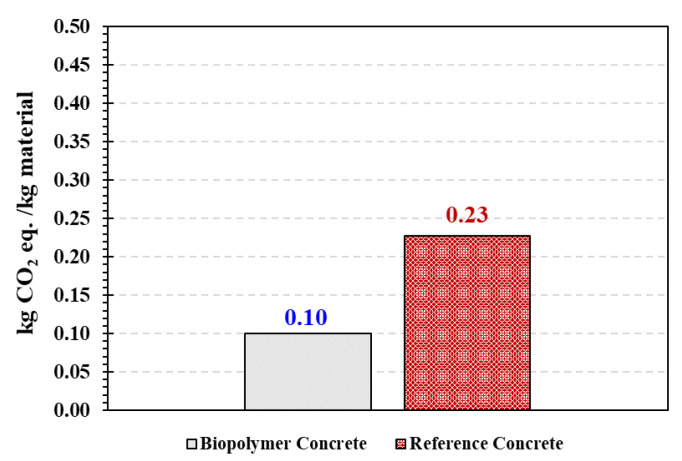
Carbon footprint analysis comparing BPC to reference cement concrete.

The carbon footprint analysis of BPC compared with the reference cement concrete is presented in Figure 16. Utilizing bio-based polyol in manufacturing BPC mixes reduced the carbon footprint by 50% compared with the reference cement concrete. In calculating the carbon footprint of BPC, the highest carbon footprint was attributed to the use of isocyanate. Therefore, utilizing bio-based isocyanate could further reduce the carbon footprint of the produced BPC. Nevertheless, utilizing BPC in applications in the construction industry where lightweight, fast setting, and high durability is required could significantly reduce the carbon footprint.

## 5. Conclusions

This paper described the development and characterization of a low-carbon footprint BPC, where bio-based PU is used as the binder in lieu of cement, modified with benzoic acid and carboxyl-functionalized multi-walled carbon nanotubes (MWCNTs). The mechanical performance, durability, and the microstructure of BPC were characterized. Chemical characterization of the bio-based PU was also performed to understand the roles of the additives in enabling a bio-based polymer that can be used to produce concrete. The following conclusions can be drawn:

- Due to the unique PU reaction, typical challenges in producing BPC include excessive foaming, fast setting, and low to no flowability.
- Benzoic acid increased the setting time from 5 min to 30 min and eliminated the excessive foaming formation. Yet, the flowability was not improved, and BPC mixes showed 0% flowability.
- The new BPC showed relatively low density, appreciable compressive strength ranging from 20–30 MPa based on the method of curing, and good tensile strength of 4 MPa. BPC had a relatively low modulus of elasticity and high Poisson’s ratio compared with cement concrete but in the same range of values reported for other PCs (e.g., polyester concrete). However, BPC outperformed ordinary concrete’s durability against aggressive environments and showed minimum weight loss while serving in highly aggressive acid environment.
- The low elastic modulus shall allow low tensile stress buildup from restrained shrinkage, which in turn, suppresses the reflective cracking.
- Chemical and microstructural analysis of bio-based PUs and PU concrete revealed that employment of MWCNTs in the polymer matrix compensated the lower crosslinking density due to the addition of benzoic acid to the PU, promoting the formation of urea bond structure, leading to further polymerization/crosslinking, and generated a percolated network structure within the polymer matrix.
- MWCNTs addition led to superior mechanical, thermal, and durability properties for the PU specimen containing benzoic acid and chemically treated MWCNTs.
- The carbon footprint for BPC was 50% lower than the reference cement concrete, which suggests BPC can provide a sustainable concrete alternative in infrastructural applications.

The produced BPC with good physical and mechanical properties, including high thermal stability and excellent durability characteristics, suggest BPC be an excellent repair material for rapid repair applications in aggressive environments or applications where a fast-curing material is required. Limitations associated with bio-polymer concrete include the low flowability of the final concrete and the limited commercial availability of bio-based polyurethane.

## Figures and Tables

**Table 1:** Properties of multi-walled carbon nanotubes (MWCNTs) (Cheap Tubes, Inc., Grafton, VT, USA).

MWCNT Properties	Measurements
Outer diameter, nm	10–20
Inner diameter, nm	3–5
MWCNTs ash, wt.%	<0.1
Purity, wt.%	>99.9
Length, µm	10–30
Specific surface area, m^2^/g	100
Bulk density, g/cm^3^	2.1
Electrical conductivity, S/cm	>10

**Table 2:** Mixture design of BPC.

MIX ID	Polyether Polyol	Polyisocyanate	Aggregate	Benzoic Acid	MWCNTs
BPC	92.0	84.6	2250.3	7.1	0.88

Ingredients represented in kg/m^3^.

**Table 3:** Flowability and setting time for BPC.

Fresh Properties	
Flowability (%)	0
Setting time (mins)	30

**Table 4:** Mechanical Properties for BPC mixes.

Mechanical Properties	BPC-A		BPC-H	
	Mean	STDV. (COV%)	Mean	STDV. (COV%)
Hardened density, ρ (kg/m^3^)	1830.0	45.0 (2.5)	1858.0	36.0 (1.9)
Modulus of elasticity, E (GPa)	9.3	0.9 (10.1)	9.9	0.9 (8.8)
Poisson’s ratio, ν	0.24	0.0 (9.8)	0.26	0.0 (4.4)

## Data Availability

The data presented in this study are available on request from the corresponding author.
